# Variations of nitrous oxide procurement per public hospital bed between Australian states and territories: A cross-sectional analysis

**DOI:** 10.1177/0310057X241262796

**Published:** 2024-08-30

**Authors:** Elizabeth P Hu, Hayden Burch, Eugenie Kayak, Forbes McGain

**Affiliations:** 1 Department of Critical Care, 2281 Melbourne Medical School, the University of Melbourne, Parkville, Melbourne, Victoria, Australia; 2 Department of Anaesthesia, Austin Health, Heidelberg, Melbourne, Victoria, Australia; 3 Departments of Anaesthesia and Intensive Care, 95317 Western Health, Footscray, Melbourne, Victoria, Australia

**Keywords:** Nitrous oxide, greenhouse gases, environment and public health, global warming

## Abstract

We aimed to identify variations in nitrous oxide (N_2_O) procurement between Australian states and territories per public hospital bed by undertaking a cross-sectional analysis of N_2_O procurement data for all Australian public hospitals from 1 January 2017 to 30 June 2022. Data were obtained from state and territory departments of health. All Australian public hospitals across six states and two territories were included. We obtained N_2_O procurement data from Department of Health representatives from all states and territories, accounting for all 697 Australian public hospitals and four public dental hospitals. The main outcome measured in this study was N_2_O procurement per public hospital bed by state or territory. Across the 5 years (1 January 2017 to 30 June 2022) an average of 242,054 (standard deviation (SD) 16,222) kg of N_2_O, equivalent to 64,144 (4299) tonnes of carbon dioxide emissions (CO_2_e), were procured per annum. CO_2_e emissions from N_2_O purchase varied more than threefold per public hospital bed between different states/territories (0.47–1.48 CO_2_e tonnes per hospital bed). There were significant variations in N_2_O procurement between Australian states and territories when adjusted for public hospital bed numbers. Further analysis of this variation to determine cause and to guide mitigation interventions is therefore warranted.

## Introduction

Increasing extreme weather events fuelled by climate change harm health.^1^ The Australian healthcare system has a significant carbon footprint, estimated at over 7% (35,772 kt of CO_2_ equivalent (CO_2_e) emissions) of the national total.^2^

Nitrous oxide (N_2_O) is used predominantly in obstetrics, anaesthesia and in the emergency department (primarily for paediatric procedures).^3^ N_2_O is a greenhouse gas that has a global warming potential approximately 265 times that of CO_2_.^4^ In obstetrics, N_2_O is used by over 40% of all women in labour in Australia.^5^ However, N_2_O administration in anaesthesia is becoming increasingly uncommon, with two-thirds of Australian anaesthetists administering N_2_O in <20% of general anaesthetics.^6^ Despite this, N_2_O’s contribution to anaesthetic emissions is likely to remain significant.^7,8^ As outlined in the recently released (December 2023) National Climate and Health Strategy, in the 2020/2021 financial year, medical N_2_O use contributed 300 kt CO_2_e emissions.^7,8^ This was equivalent to 20% of the total Australian health system’s Scope 1 CO_2_e emissions, according to National Greenhouse Accounts data.^7,8^ In England, National Health Service (NHS) CO_2_e emissions from N_2_O have been estimated to account for 1.5% to 2% of national healthcare CO_2_e emissions, with N_2_O contributing to approximately 75% of all inhaled anaesthetic emissions.^9^ Similarly, Irish and Austrian data found N_2_O’s contribution to inhaled anaesthetic emissions to be over 84% and 91% respectively.^10,11^ However, the proportion of healthcare’s carbon footprint could be addressed by reducing or even eliminating inhaled anaesthetic gases.^12,13^

Several studies have found a large discrepancy between the purchased volume and the clinically utilised volume of N_2_O, which is attributed to N_2_O infrastructure leakage.^3,14^ Current published data have shown that one Australian hospital had an estimated 77% of all purchased N_2_O leaked prior to clinical administration,^14^ with 84% estimated at another hospital.^15^ Prior United Kingdom (UK) studies found similar proportional N_2_O leakage from hospital pipe infrastructure.^9^

In accordance with the Intergovernmental Panel on Climate Change (IPCC) 2015 Paris Agreement, Australia, along with several other countries, committed to net zero carbon emissions by 2050.^16,17^ Emissions from medical N_2_O are minor from a global perspective, contributing between 1% and 3% of N_2_O’s global total,^18^ however, to address healthcare’s contribution to the national carbon footprint, data on key contributors (i.e. carbon hotspots) are needed. Australian medical N_2_O emissions may be an identified healthcare carbon hotspot. However, whilst presently N_2_O is a reportable gas under the Australian National Greenhouse and Energy Reporting Scheme,^19^ healthcare data are difficult to access. We hypothesised that Australian public healthcare procurement over the 5-year period would be stable, and similar across all states and territories when accounting for hospital beds.

## Methods

We undertook a national, cross-sectional study of annual N_2_O procurement data for Australian healthcare. We sought data for the 5-year period 2017–2022 from four Australian sources. We initially contacted the following three sources: (i) the Federal Department of Agriculture, Water, and Environment, and ultimately the Department of Industry, Science, and Resources, (ii) commercial N_2_O gas suppliers BOC Linde, Air Liquide, Coregas, and the Australia New Zealand Industrial Gas Association, and (iii) multiple private hospital groups (e.g. Epworth, St John of God, Healthscope, UnitingCare, St Vincent’s Private and Mercy Health). However, our data requests to these three sources were either declined due to privacy concerns (except for St John of God) or not responded to despite multiple attempts.

We then sought and received data by email correspondence from the six state and two territory governments via the Australasian Health Infrastructure Alliance and from Department of Health representatives. Inclusion criteria were all Australian state and territory public hospital N_2_O or CO_2_e data from the period beginning 1 January 2017. There were no exclusion criteria. These amalgamated hospital N_2_O procurement data included all 697 Australian public hospitals as well as four public dental hospitals.

The study outcomes measured were the total N_2_O procurement data in kilograms (kg) and its CO_2_e emissions in kg, including a per public hospital bed estimate. All data were provided by Department of Health representatives. Victoria provided procurement data in tonnes CO_2_e, whilst all other states and territories provided procurement data in kg of N_2_O. To convert data between N_2_O and CO_2_e we used 1 kg N_2_O = 265 kg CO_2_e, as per the 2023 Australian National Greenhouse Accounts Factors.^4^ Entonox was calculated as 56.7% N_2_O, 43.3% oxygen by weight. Five years of N_2_O procurement data were collected and annualised for each state and territory.

States and territories provided data in either financial year or calendar year format. For Victoria, New South Wales, South Australia and the Australian Capital Territory (ACT), financial year data were provided from 1 July 2017 to 30 June 2022.

Queensland, Western Australia (WA), Tasmania, and the Northern Territory provided calendar year data from 1 January 2017 to 31 December 2021. Data were provided in annualised format only, hence presenting data for all states and territories for either financial year or calendar year periods was not possible. Data prior to 2017 were unavailable for most states and territories.

Data were obtainable from only one private hospital group, St John of God, for three financial years (2019–2022). All other private organisations/companies contacted either declined our data request or did not correspond, despite at least two requests to each. Therefore, the St John of God data were not included in our analysis.

We calculated the CO_2_e emissions for the number of Australian cars on the road annually by dividing our total N_2_O amount represented as CO_2_e by the conversion factor for CO_2_ emissions per kilometre (146.5 g) and the estimated distance driven by a vehicle in Australia annually (12,100 km).^20,21^

We estimated the CO_2_e from N_2_O procurement per public hospital bed for each state and territory with data from the 2020–2021 hospital resources report from the Australian Institute of Health and Welfare.^22^

Ethical approval was obtained from Western Health Office for Research prior to study commencement (reference number QA2022.38).

## Results

**Figure 1.**
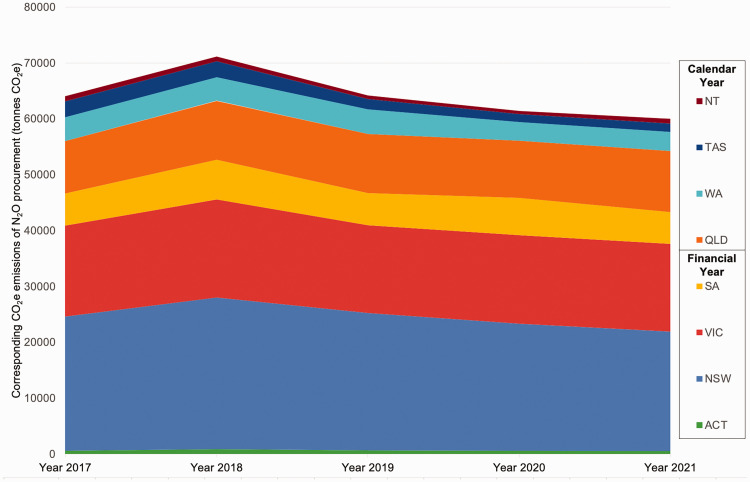
Carbon dioxide equivalent emissions (CO_2_e) of national procurement of nitrous oxide (N_2_O) in Australian public healthcare for a 5-year period. *Note*: Victoria (VIC), New South Wales (NSW), South Australia (SA) and the Australian Capital Territory (ACT) provided financial year data (1 July 2017 to 30 June 2022). Queensland (QLD), Western Australia (WA), Tasmania (TAS), and the Northern Territory (NT) provided calendar year data (1 January 2017 to 31 December 2021).

The total Australian national public hospital mean annual N_2_O procurement was 242,054 kg (SD 16,222) between 1 January 2017 and 30 June 2022. This N_2_O purchase was equivalent to 64,144 tonnes CO_2_e emissions per annum (SD 4299). Figure 1 provides a visual representation of state/territory quantitative distribution as well as procurement over time.

**Figure 2.**
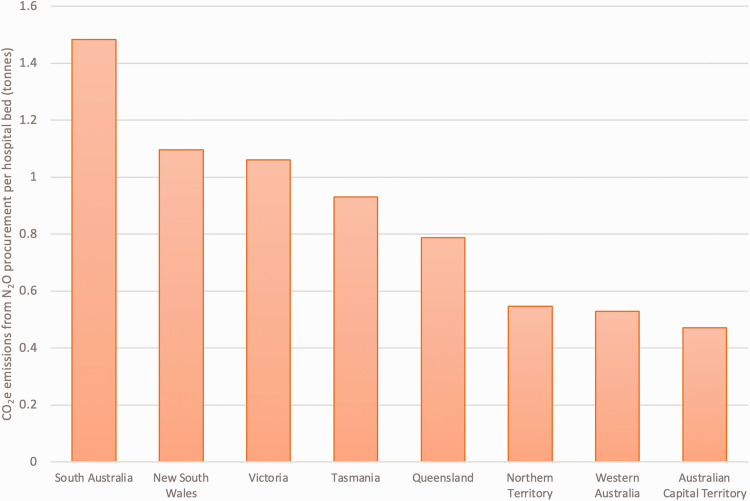
State/territory carbon dioxide equivalent (CO_2_e) emissions from nitrous oxide (N_2_O) procurement per hospital bed, 2020 (results include amalgamated financial year and calendar year data).

For the year 2020, we calculated CO_2_e from N_2_O procurement per public hospital bed for each state/territory (Table 1), with a threefold variation in the N_2_O procurement per annum between states and territories per public hospital bed (Table 1, Figure 2). A yearly breakdown of procurement data including CO_2_e emissions per state/territory are provided in Tables 2 and 3.

**Table 1.** State/Territory carbon dioxide equivalent (CO_2_e) emissions from nitrous oxide (N_2_O) procurement per public hospital bed, 2020–2021 (results include amalgamated financial year and calendar year data.

State/territory	N_2_O in CO_2_e (tonnes) in 2020	Public hospital beds	N_2_O in CO_2_e purchased per hospital bed (tonnes)
South Australia	6695	4514	1.48
New South Wales	22,791	20,787	1.10
Victoria	15,811	14,913	1.06
Tasmania	1472	1583	0.93
Queensland	10,255	13,032	0.79
Northern Territory	585	1072	0.55
Western Australia	3297	6243	0.53
Australian Capital Territory	560	1189	0.47

**Table 2.** New South Wales, Victoria, South Australia and Australian Capital Territory public hospital N_2_O procurement (kg) and corresponding CO_2_e (tonnes), 2017–2021 financial years.

Year	2017	2018	2019	2020	2021	Mean annual (standard deviation)	Five-year total
New South Wales N_2_O kg	90,643	102,501	92,705	86,002	80,483	90,467 (8208)	452,334
New South Wales kg N_2_O CO_2_e	24,020	27,163	24,567	22,791	21,328	23,974 (2175)	119,869
Victoria N_2_O kg	61,589	66,125	59,275	59,664	59,317	61,194 (2916)	305,970
Victoria kg N_2_O CO_2_e	16,321	17,523	15,708	15,811	15,719	16,216 (773)	81,082
South Australia N_2_O kg	21,609	26,938	21,577	25,264	21,447	23,367 (2566)	116,835
South Australia kg N_2_O CO_2_e	5,726	7,139	5,718	6,695	5,683	6192 (680)	30,961
Australian Capital Territory N_2_O kg	2113	3312	2577	2115	2112	2446 (524)	12,229
Australian Capital Territory kg N_2_O CO_2_e	560	878	683	560	560	648 (139)	3241
New South Wales, Victoria, South Australia and Australian Capital Territory combined total N_2_O kg	175,954	198,876	176,134	173,045	163,359	177,474 (13,048)	887,368
New South Wales, Victoria, South Australia and Australian Capital Territory combined total kg N_2_O CO_2_e	46,628	52,702	46,676	45,857	43,290	47,030 (3458)	235,152

*Note*: N_2_O: nitrous oxide; CO_2_e: carbon dioxide equivalent; kg: kilograms.

Victorian Department of Health representatives used a global warming potential (GWP) of 300 (i.e. 1 kg of N_2_O = 300 kg of CO_2_e), whilst our calculations used GWP 265, based on the latest 2023 National (Australian) Greenhouse Accounts Factors.

**Table 3.** Queensland, Western Australia, Tasmanian and Northern Territory public hospital N_2_O procurement (kg) and corresponding CO_2_e (tonnes), 2017–2021 calendar years.

Calendar year	2017	2018	2019	2020	2021	Mean annual (standard deviation)	Five-year total
Queensland N_2_O kg	35,404	39,709	40,007	38,699	41,381	39,040 (2247)	195,200
Queensland N_2_O CO_2_e	9665	10,841	10,922	10,565	11,297	10,658 (614)	53,290
Western Australia N_2_O kg	16,159	15,856	16,705	12,440	12,848	14,802 (1998)	74,008
Western Australia N_2_O CO_2_e	4411	4329	4561	3396	3507	4041 (546)	20,204
Tasmania N_2_O kg	10,701	10,948	6901	5391	5653	7919 (2715)	39,594
Tasmania N_2_O CO_2_e	2921	2989	1884	1472	1543	2162 (741)	10,809
Northern Territory N_2_O kg	3344	3101	2384	2206	3065	2820 (495)	14,100
Northern Territory N_2_O CO_2_e	913	846	651	602	837	770 (135)	3849
Queensland, Western Australia, Tasmanian and Northern Territory combined N_2_O kg	65,589	69,602	65,985	58,725	62,933	64,567 (4038)	322,834
Queensland, Western Australia, Tasmanian and Northern Territory combined N_2_O CO_2_e	18,823	19,005	18,018	16,035	17,184	17,813 (1229)	89,065

*Note*: N_2_O: nitrous oxide; CO_2_e: carbon dioxide equivalent; kg: kilograms.

Public hospitals in the more populous states of NSW and Victoria combined procured 63% of national public hospital N_2_O (452,334 (37.4%) and 305,970 (25.3%) of 1,210,270 kg total during the approximated 5-year period between 1 January 2017 to 30 June 2022).

## Discussion

The mean annual N_2_O purchased by Australian public hospitals was calculated to be equivalent to the annual carbon emissions of over 36,000 Australian cars on the road.^19,20^ Although our calculated CO_2_e emissions from public hospital N_2_O procurement may appear low in isolation, establishing baseline N_2_O procurement data and subsequent CO_2_e emissions data are important to guide cumulative efforts from all feasible interventions to reduce Australian healthcare’s significant CO_2_e emissions.

Public hospital procurement of N_2_O per public hospital bed varied considerably between different states and territories, with SA purchasing approximately three times more N_2_O than WA and ACT per number of public hospital beds.^22^ Our discussions and verifications with state healthcare representatives suggested the reason for variation in N_2_O procurement was probably multifactorial, including infrastructure leakage, use in maternity services, paediatric services, and clinician preference. However, we speculate that the most likely reason for this variance is infrastructure leaks. In SA, where procurement is greater than any other state/territory per hospital bed, our correspondence with the South Australian Department of Health representatives revealed significant leakage concerns. Restrictions to N_2_O administration during the COVID-19 pandemic were documented in SA, but did not lead to any reductions in procurement.^23^ Likewise, on a national level, between 2021 and 2022, there was a 17% decrease in elective surgery admissions from public hospital wait lists compared with 2017/2018 data.^24^ However, national N_2_O procurement data remained relatively stable during these periods, suggesting that procurement is unlikely to be driven primarily by clinical administration.

Our rationale that leakage is the likely reason for the variation between jurisdictions, while requiring confirmation with further investigation, is supported by previous reports of leakage from faulty N_2_O infrastructure being demonstrated to significantly increase over-procurement of N_2_O relative to clinical use both internationally and in Australia.^9,13,14^ Published studies from Western Health and Alfred Health have estimated leakage of between 77% and 84% in observational studies.^14,15^ This is also reflected in UK studies where leakage rates of up to 98% were found in NHS Scotland.^9^ We are also aware from personal communications that hospitals in SA and WA have recorded significant leakage from N_2_O infrastructure. Accordingly, as advocated for by the UK-based Nitrous Oxide Project, who have confirmed nationwide leakage as an issue, a multidisciplinary approach incorporating accurate monitoring of procurement and consumption data by all hospitals and healthcare services is paramount to tackling N_2_O wastage and instilling responsible stewardship.^9,25,26^ Novel and cost-effective methods of assessing N_2_O leaks such as weighing gas cylinders and comparing usage from electronic medical records could be used to detect and quantify leakage.^15^ Clinicians and health leaders advocating for the removal of extensive piped supply of N_2_O within the master plan for new hospital developments will be crucial for waste prevention.^13,25,26^ Similarly, decommissioning N_2_O manifolds in hospitals that do not provide maternity care and transitioning to canister storage close to the point of clinical care will be key in addressing infrastructure leakage and reducing emissions.^9,13,25^

This is the first cross-sectional analysis of medical N_2_O procurement in Australian healthcare. The UK, the Republic of Ireland, and Austria have transparent and available N_2_O emissions data.^9–11^ The purchase of N_2_O in England potentially contributes to 1.5%–2% of NHS England’s total carbon footprint^9^; however, in Australia, we were unable to accurately calculate the contribution of total purchased N_2_O to healthcare’s carbon footprint due to poor data transparency from the private health sector. Measurement and benchmarking of greenhouse gas emissions from the entire Australian healthcare sector are required to inform clinical practices, infrastructure decisions and healthcare policy.^12^ Therefore, improved access to comprehensive Australian healthcare procurement data is both urgent and integral to assisting the transition to low-carbon healthcare.

Our study was limited by obtaining public hospital data only. Private and public–private partnership hospitals may have alternate procurement processes with separate data not available to state/territory Department of Health representatives. Private sector N_2_O procurement may be substantial, and our results may not be generalisable to national private sector procurement. Given that 25.4% of births occur in private hospitals in Australia,^5^ the total Australian healthcare N_2_O emissions will be higher than our estimate, which only includes public hospital procurement. Hence, further investigation and access to nationwide N_2_O data are required to determine the private sector’s contribution to N_2_O emissions from Australian healthcare, including dentistry. There is also potential imprecision in the data comparison as data were obtained in either financial year or calendar year format.

All Australian state, territory, and federal governments have pledged to meet carbon emission reduction targets by 2030–2050.^27^ For Australian healthcare to contribute meaningfully towards the targets, coordinated, committed abatement strategies are needed.^12,25,26^ Clinicians have an important role to play in the mitigation of N_2_O, as well as volatile anaesthetic greenhouse gases and respiratory inhaler propellants, which, unless addressed, will increase proportionately to healthcare’s total CO_2_e emissions as energy grids and supply chains decarbonise.^12,26,27^

The recent (December, 2023) publication of Australia’s first National Health and Climate Strategy (National Strategy) provides an important framework for standardised metrics and monitoring of national greenhouse gas data, inclusive of N_2_O.^7,8^ The National Strategy sourced data for medical N_2_O use from the Department of Climate Change, Energy, the Environment and Water, in which healthcare came under Accommodation, Food Services, Education and Health Services (including public, private, and dental care).^19^ A discrepancy between the annual medical N_2_O use from the National Strategy (300 kt CO_2_e) and our calculations (65 kt CO_2_e, i.e. approx. 20%)^8^ is to be expected due to different inclusion criteria. However, efforts should be made to robustly gather data (as occurred in our study) for all sources of medical N_2_O use, to carefully analyse the N_2_O emissions from healthcare, including private and dental healthcare use.

We found it challenging to obtain N_2_O procurement data, despite its clinical and environmental importance for the sector. Coordinated systemwide approaches such as the Nitrous Oxide Project and the Greener NHS Plan have proven to be effective in the UK^9,25,26^ and provide a template for policymakers and healthcare leaders in Australia.

## Conclusion

We quantified variations in N_2_O procurement per bed number between states and territories in Australian public healthcare. We revealed inadequacies in access to granular national data for N_2_O procurement, including access to private hospital and dental procedure data. Our study adds weight to the importance of local data to inform clinicians and healthcare institutions about the carbon footprint of healthcare, as in the UK, the Republic of Ireland, and Austria where selected clinicians have access to N_2_O data and can promptly respond to variabilities in procurement that do not correlate with clinical administration. We recommend clinicians and representatives to investigate and address infrastructure leakage that may be driving procurement despite low clinical utilisation. Access to more detailed procurement and usage data is vital in advocating for evidence-based emissions reduction.
